# Insights into Local Tumor Microenvironment Immune Factors Associated with Regression of Cutaneous Melanoma Metastases by *Mycobacterium bovis* Bacille Calmette–Guérin

**DOI:** 10.3389/fonc.2017.00061

**Published:** 2017-04-05

**Authors:** Junbao Yang, Maris S. Jones, Romela Irene Ramos, Alfred A. Chan, Agnes F. Lee, Leland J. Foshag, Peter A. Sieling, Mark B. Faries, Delphine J. Lee

**Affiliations:** ^1^Dirks/Dougherty Laboratory for Cancer Research, Department of Translational Immunology, John Wayne Cancer Institute, Providence Saint John’s Health Center, Santa Monica, CA, USA; ^2^Division of Surgical Oncology, John Wayne Cancer Institute, Providence Saint John’s Health Center, Santa Monica, CA, USA; ^3^Division of Dermatology, Los Angeles Biomedical Research Institute, Harbor-UCLA Medical Center, Torrance, CA, USA; ^4^Melanoma Research Program, John Wayne Cancer Institute, Providence Saint John’s Health Center, Santa Monica, CA, USA; ^5^Translational Immunology, NantBioscience, Inc., Culver City, CA, USA

**Keywords:** bacille Calmette–Guérin, melanoma, γδ T cells, immunotherapy, tumor microenvironment

## Abstract

*Mycobacterium bovis* bacille Calmette–Guérin (BCG) is listed as an intralesional (IL) therapeutic option for inoperable stage III in-transit melanoma in the National Comprehensive Cancer Network Guidelines. Although the mechanism is unknown, others have reported up to 50% regression of injected lesions, and 17% regression of uninjected lesions in immunocompetent patients after direct injection of BCG into metastatic melanoma lesions in the skin. BCG and other mycobacteria express ligands capable of stimulating the γ9δ2 T cells. Therefore, we hypothesized that γ9δ2 T cells play a role in promoting BCG-mediated antitumor immunity in patients treated with IL-BCG for in-transit cutaneous melanoma metastases. Indeed, we found γ9δ2 T cell infiltration in melanoma skin lesions during the course of IL-BCG treatment. Gene expression analysis revealed that BCG injection elicits the expression of a vast array of chemokines in tumor lesions, including strong expression of CXCL9, 10, and 11, a set of chemokines that attract T cells expressing the CXCR3 chemokine receptor. In corroboration with our hypothesis, approximately 85% of γδ T cells express high levels of CXCR3 on their surface. Importantly, the injected tumor lesions also express genes whose protein products are the antigenic ligands for γδ T cells (BTN3A1 and MICB), and the cytokines that are the typical products of activated γδ T cells. Interestingly, we also found that γδ T cells infiltrate the regressed lesions that did not receive BCG injections. Our study suggests that γ9δ2 T cells may contribute to melanoma regression induced by IL-BCG treatment.

## Introduction

Melanoma is a cutaneous malignancy that kills ~10,000 people annually in the US (1). In contrast to most other types of cancer in which incidences are steadily declining, the incidence of melanoma continues to climb, especially in young patients (1). Melanoma treatment has been strikingly improved through the use of immune-based approaches (2–4), emphasizing the significant protective role of immune cells against melanoma. Intralesional (IL) *Mycobacterium bovis* bacille Calmette–Guérin (BCG), or IL-BCG, is currently a recommended therapy in the National Comprehensive Cancer Network (NCCN) Guidelines (version 1.2017) for inoperable stage III in-transit metastatic melanoma. Direct injection of BCG into metastatic melanoma lesions in the skin has resulted in up to 90% regression of injected lesions and 17% regression of uninjected lesions in immunocompetent patients (5, 6). Epidermal injection of BCG induces a typical delayed hypersensitivity response, characterized by numerous chemokines and cytokines and recruitment of a vast array of immune cells into the BCG injected sites (7). It is well accepted that the immune response plays an important role in IL-BCG-induced tumor regression.

Mycobacterial infection recruits γδ T cells to the infected lymph nodes during early stages of infection (8). This rapid response by γδ T cells is an important factor in the host control of the mycobacterial infection (9, 10). γδ T cells are a subset of T cells making up 1–5% of the total CD3+ T cells in peripheral blood (11, 12). Unlike αβ TCR lineage, γδ T cells usually do not express CD4 or CD8, but only CD3 (11, 13), and direct TCR signaling through the associated CD3 complex (14). γδ T cell antigen recognition does not require conventional antigen presentation in the context of MHC (15), and they may recognize unconventional antigens including stress molecules like MICA and MICB and non-peptide metabolites of isoprenoid biosynthesis (16–19). Human γδ T cells can be divided into three main populations based on their δ chain expression: Vδ1, Vδ2, and Vδ3. γδ T cells expressing Vδ2 chain represent the majority of circulating γδ T cells in healthy human adults, comprising 50–90% of the peripheral γδ T cell population. The Vδ2 chain pairs almost exclusively with Vγ9. Stimulation of PBMCs with BCG induces the preferential expansion of the γ9δ2 subset (20, 21). This subset of γδ T cells recognizes small non-peptidic phosphorylated antigens, such as microbial hydroxymethyl-but-2-enyl-pyrophosphate (HMBPP) produced by infectious agents and recognized as non-self, and induces the production of host phosphoantigens, such as isopentenyl pyrophosphate (IPP), the intermediates of isoprenoid biosynthesis of mevalonate pathway, accumulated in infected or transformed cells (19, 22–25). Mycobacteria can produce HMBPP and stimulate host cells to produce IPP (18). Tumor cells themselves can also express IPP to the levels sufficient to activate Vδ2 T cells (26, 27) due to the dysfunctional mevalonate pathway in tumors (28). Therefore, γδ T cells not only participate in the control of mycobacterial infections but have also displayed a broad cytotoxicity against a wide variety of tumor types, including leukemia, neuroblastoma, melanoma, and various carcinomas (29–33). Besides their direct tumoricidal activity, activated γδ T cells also display antigen-presenting cell properties characterized by the expression of high levels of HLA-DR and costimulatory molecules such as CD80 and CD86 (34, 35) that could support adaptive immune responses (36). With all these properties, γδ T cells have been considered and evaluated as adoptive immunotherapeutic cells for tumor immunotherapy (37–39).

In this study, we investigate whether γδ T cells play a role in IL-BCG-induced melanoma regressions.

## Materials and Methods

### Patients and IL-BCG Treatment

Between 2011 and 2013, eight patients studied were diagnosed with stage III in-transit melanoma and treated with IL-BCG according to the NCCN guidelines. Patient information was obtained retrospectively from a single, high volume melanoma referral center following internal review board (IRB) approval. The goal of treatment was local control and palliation or prevention of symptoms associated with in-transit disease. Following a negative tuberculin skin test result, patients were sensitized to BCG at distant sites with intradermal injections of BCG to sites adjacent to regional nodal basins initially. Following sensitization, if applicable, patients then received intradermal BCG injections in the target in-transit lesions, typically 2 weeks later. IL injection doses following sensitization were typically lower and titrated to produce a moderate local inflammatory response to avoid skin necrosis and the development of vesicles. These in-transit metastases were injected no more frequently than every 2 weeks. IL-BCG administration was continued until complete resolution or disease progression. The use of patient and healthy donor samples was reviewed and approved by Alpha IRB in this study.

### Immunohistochemistry

The melanoma specimens were obtained by surgical resection or open biopsy. Frozen melanoma sections (5-µm thick) were fixed in pre-cooled acetone and rinsed with phosphate buffered saline at a neutral pH. The tissue sections were blocked with normal horse serum. Properly diluted 100 µL of anti-CD3 (F7.2.38, Dako), γδ TCR (IMMU510, Fisher Scientific), Vγ9 TCR (7A5, Fisher Scientific), or isotype control antibodies were applied on tissue sections. Anti-mouse IgG–HRP conjugate was used as a secondary antibody to develop the AEC color of specific staining.

### RNA Isolation, cDNA Preparation, and RNA Sequencing (RNAseq)

Bacille Calmette–Guérin injected and uninjected melanoma biopsies from IL-BCG-treated patients were minced into small pieces. Total RNA from both sources was extracted using the Qiagen RNeasy mini kit (Qiagen, Valencia, CA, USA). cDNA libraries were prepared using TruSeq RNA Library Preparation Kit v2 (RS-122-2001) according to the “TruSeq RNA Sample Preparation v2 Guide” (Illumina, San Diego, CA, USA). Briefly, poly-A+ mRNA was isolated from 300 ng total RNA using polyoligo-dT attached on magnetic beads. After fragmentation with divalent cations under elevated temperature, the cleaved RNA fragments were transcribed into first-strand cDNA using reverse transcriptase and random primers. Second-strand cDNA was synthesized using DNA polymerase I and RNase H. These cDNA fragments were end-repaired, added with single “A” base, and ligated with adapters. The products were then purified and enriched with PCR to create the final cDNA library. Libraries were sequenced on Illumina HiSeq2000 at 50 million reads per sample and 1 × 50 read length. These procedures were performed by the UCLA Clinical Microarray Core facility.

### RNAseq Data Processing and Analysis

The reads obtained by RNAseq were processed and analyzed with specific tools piped together using Ubuntu. Quality assurance of reads (GC content, adaptors, and PHRED score assessment) were done with FastQC. Trimming to remove poor quality reads and adapters was performed using Trimmomatic. The reads were scanned with a four base wide sliding window and cut when average quality dropped below 15 phred score. Reads less than 36 bases long were removed. Read-mapping to the human reference genome hg19 and abundance estimation of genes and isoforms was done using Bowtie2 aligner within RSEM with default values (40). We compared the RSEM normalized RPKM/FPKM values of chemokine receptors, cytokines, butyrophilin subfamily 3 member A1 (BTN3A1), and MHC class-I polypeptide-related sequence A and B (MICA and MICB). Vγ9 was quantitated by the basic local alignment search tool using a fasta reference downloaded from the NCBI database. The Vγ9 counts were normalized into counts per million.

### T Cell Composition and Phenotype Analysis of PBMCs

Frozen PBMCs (0.2–0.5 × 10^6^/tube) from healthy donors were stained with anti-CD3 (APC-H7, BD Biosciences), anti-αβ TCR (FITC, BioLegend), and anti-γδ TCR (APC), along with additional antibodies, Vγ9 TCR (PE), CXCR3 (CD183) (PE), CCR4 (CD194) (PerCP-Cy5.5), CCR6 (CD196) (PerCP-Cy5.5), and CLA (PE). The phenotypes of cells were analyzed on BD FACSVerse.

To study the effect of BCG stimulation on the expansion of γδ T cells, frozen PBMCs (2.5 × 10^6^/mL/well in a 48-well plate) from healthy donors were suspended in T cell culture medium (RPMI-1640, containing 10% human serum, 1% sodium pyruvate, 1% penicillin/streptomycin; Invitrogen, Carlsbad, CA, USA), stimulated with or without BCG live vaccine (TICE BCG University of Illinois, IL, USA) at final concentration of 10^5^ colony-forming units/milliliter. After 6 days in culture at 7% CO_2_, 37°C, the cells were stained with anti-CD3 (APC-H7), anti-γδ TCR (APC), along with one of the following additional antibodies: anti-αβ TCR (FITC), HLA-DR (FITC), or Vγ9 TCR (PE). The phenotypes of cells were analyzed on BD FACSVerse.

### Isolation and Composition Analysis of Tumor Infiltrated Lymphocytes (TILs)

Biopsies of regressed and non-regressed melanoma tissue were sterilely minced into pieces 1–2 mm in diameter. The minced tissue specimens were then attached to the cell foam matrices pre-coated with collagen to resemble 3D culture. The tumor specimens were cultured in 2 mL of skin T cell medium (Iscove’s medium containing 10% fetal bovine serum, 100 U/mL IL-2, and 10 ng/mL of IL-15) in a 24-well plate. The medium was replaced with half of fresh skin T cell medium every other day. After 21 days of culture, T cells that migrated out of the melanoma tissue were collected. Lymphocyte composition of TILs was analyzed with anti-γδ and αβ TCR antibodies by flow cytometry.

### Statistical Analysis

For T cell composition and phenotype analyses by flow cytometry, paired Student’s *t*-test was used as indicated in the figure captions.

For the RNAseq gene expression analysis, immune-related genes and housekeeping genes were preselected to reduce false discovery rate from multiple hypothesis testing. The differential expression between BCG injected (*n* = 9) and uninjected (*n* = 10) was tested using the non-parametric two-sided Mann–Whitney test. False discovery rate was controlled for by applying Benjamini–Hochberg adjustment to resulting *p*-values. Genes meeting the 5% alpha threshold were considered to be statistically significant. Statistics were performed on R version 3.3.1 and plots generated using ggplot2_2.2.0 package.

## Results

### IL-BCG Induces Melanoma Regression Accompanied by γδ T Cell Infiltration

**Figure 1:**
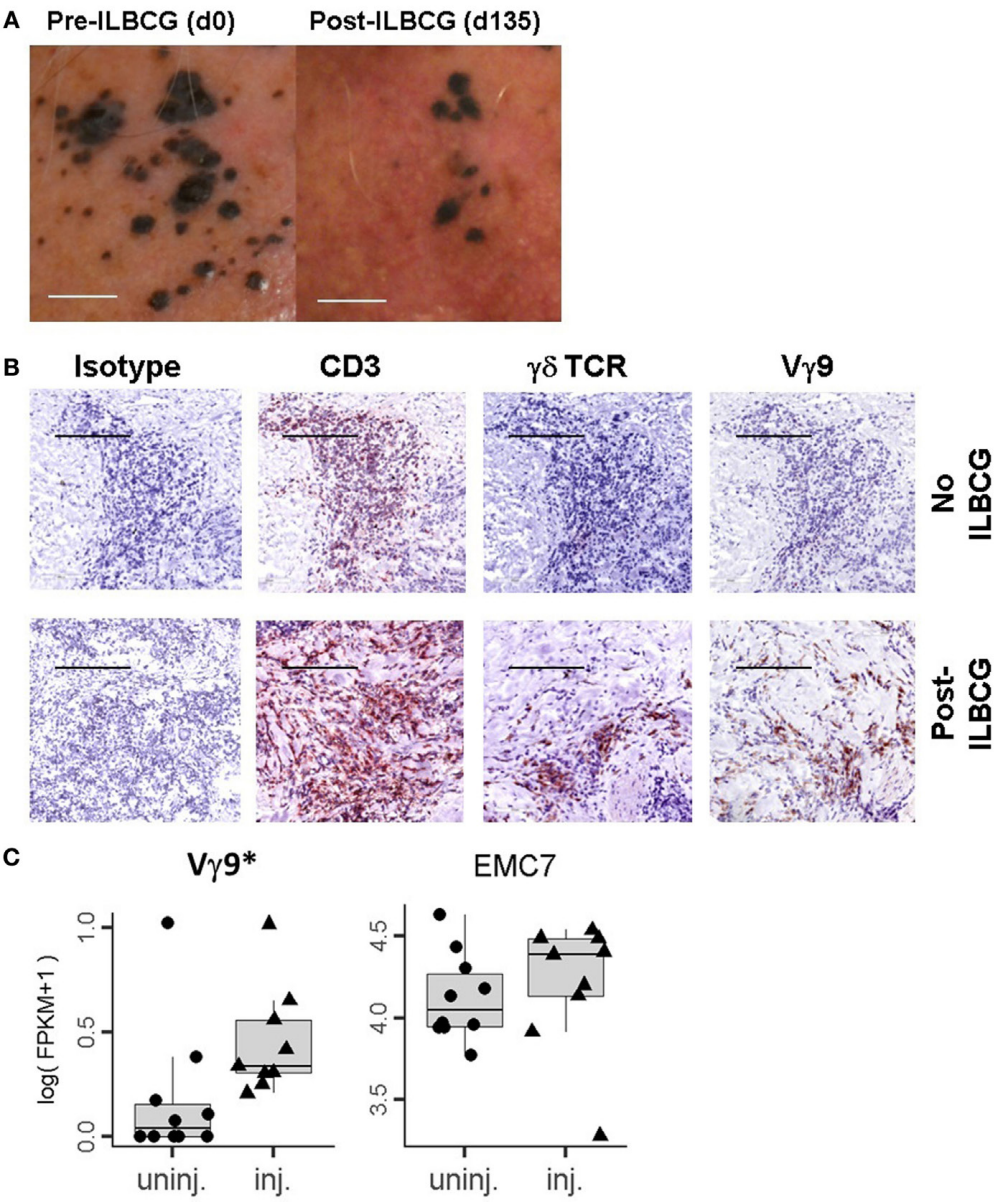
**IL-BCG-induced tumor regression is associated with the presence of γδ T cells**. **(A)** IL-BCG induces tumor regression. Scale bars represent 1 cm. **(B)** Presence of γ9 γδ T cells in IL-BCG-injected tumors. Scale bars represent 100 µm. **(C)** Increased mRNA expression of Vγ9 transcripts in the injected tumor tissue by RNA sequencing [fragments per kilobase of exon per million fragments mapped (FPKM)]. EMC7 is a representative housekeeping gene control (41).

In this study, eight in-transit stage III melanoma patients were treated with IL-BCG as described in the Section “Materials and Methods.” As expected, BCG injection induced complete or partial regression of injected melanoma lesions in six of eight patients (Figure 1A). The details of patients’ response to therapy are summarized in Table S1 in Supplementary Material. Immunohistological analysis revealed that while CD3 cell infiltration occurs in both IL-BCG-treated and non-treated lesions, γδ T cell infiltration was observed only in the IL-BCG-treated lesions (Figure 1B). Some of these infiltrated cells are Vγ9-expressing cells (Figure 1B). To verify that an influx of γδ T cells occurred with IL-BCG injection, the total RNA samples isolated from these lesions were subjected to the high-throughput RNAseq analysis. In agreement with the above finding, the Vγ9 gene expression was significantly higher in the IL-BCG-treated lesions (Figure 1C) according to RNAseq analysis. There were no differences in the expression of housekeeping genes such as ER membrane protein complex subunit 7 (EMC7), charged multivesicular body protein 2A (CHMP2A), and chromosome 1 open reading frame 43 (C1orf43) (Figure 1C and data not shown) (41).

### IL-BCG Induces Chemokines That Attract CXCR3-Expressing T Cells; γδ T Cells Are Predominantly CXCR3-Expressing Cells

**Figure 2:**
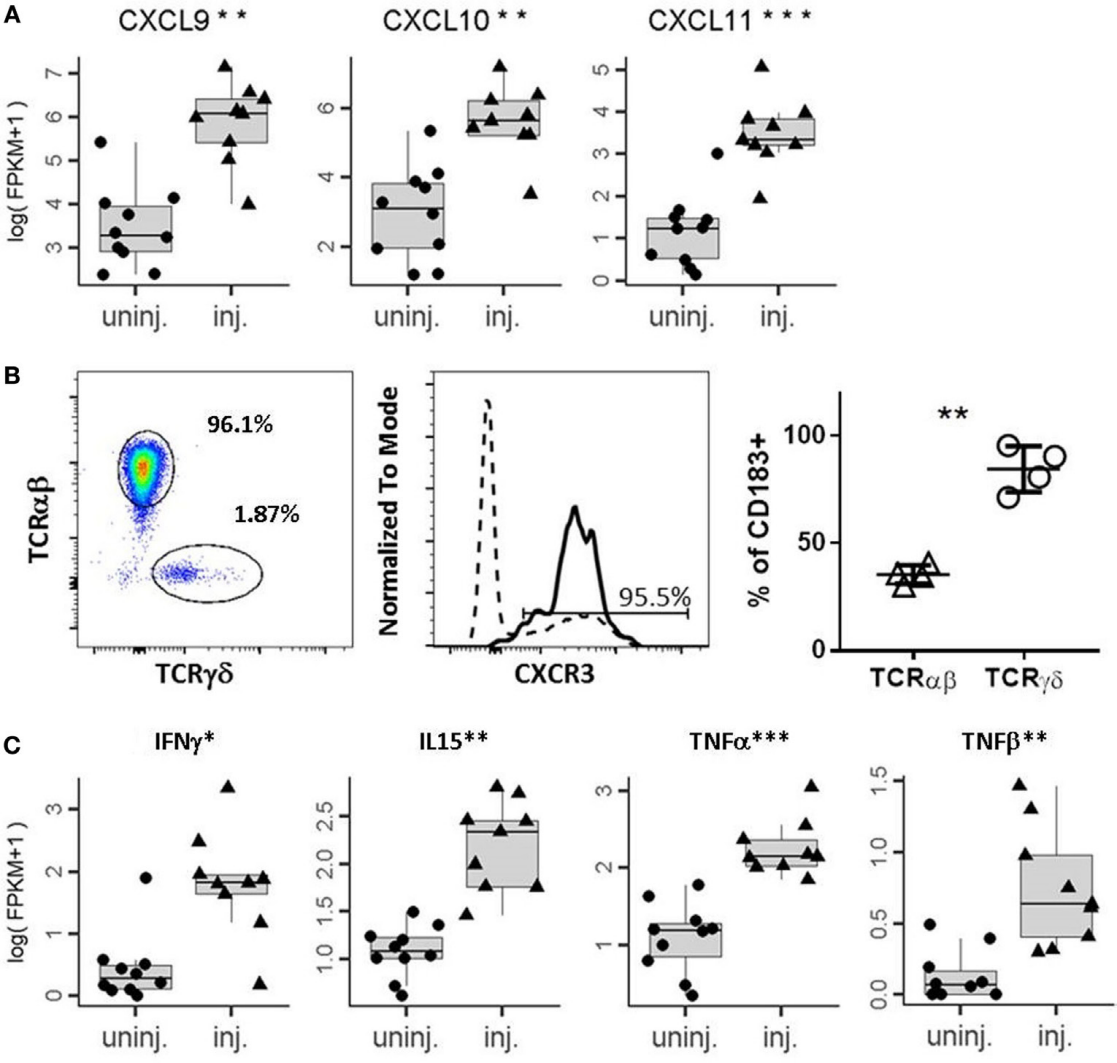
**IL-BCG induces secretion of chemokines that attract γδ T cells**. **(A)** Elevation of chemokines that attract CXCR3 expression cells. ***, Student’s *t*-test, *p* < 0.001. **(B)** γδ T cells express high levels of CXCR3. Right panel: percentage of γδ and αβ T cells among CD3+ T cells from a representative donor. Middle panel: histogram of FACS staining of CXCR3 on γδ T cells (solid line) and αβ T cells (dashed line). Right panel: summary of % of CXCR3+ T cells in αβ T cells and γδ T cells, respectively. **, paired *t*-test, *p* < 0.01. **(C)** Elevation of cytokines that can be contributed from activated γδ T cells.

Intralesional injection of BCG induces a massive secretion of chemokines and cytokines that recruit immune cells (7). We interrogated whether IL-BCG treatment induces chemokines responsible for attracting γδ T cells to injection sites using RNAseq. We found that many chemokines were significantly upregulated in the IL-BCG-treated tumors (Table 1). Among them, chemokines CXCL9, CXCL10, CXCL11 for CXCR3 (CD183), and CCL20 for CCR6 (CD196) receptors were all upregulated (Figure 2A; Figure S1A and Table S1 in Supplementary Material). Chemokines CXCL9, CXCL10, and CXCL11 for CXCR3 receptor showed the highest fold changes (approximately 10-fold) and absolute increase in gene expression (Figure 2A; Table 1). Corroborating that finding, most γδ T cells from PBMCs express high levels of CXCR3 receptors on their surfaces (Figure 2B), while very few γδ T cells express CCR4 or CCR6 receptors (Figures S1B,C in Supplementary Material). These data suggest that IL-BCG treatment could attract γδ T cells to the injection sites through the CXCL9/10/11–CXCR3 chemotaxis axis. Consistent with the attraction of γδ T cells, IL-BCG also elicits a significant elevation of signature cytokines from γδ T cells, including IFNγ (42, 43), TNFα, TNFβ, and IL-15 (44) (Figure 2C; Table 2).

**Table 1:** **Expression levels (FPKM) of chemokines in bacille Calmette–Guérin injected and uninjected melanoma tissue**.

Chemokine	avg.Uninj (10)	avg.Inj (9)	*p*.value	*p*.adjusted	Significance
CCL1	0	0.7	---	---	
CCL2	40.8	157.1	0.000	0.000	***
CCL3	12.4	75.7	0.001	0.005	**
CCL3L1	1.8	9.6	0.082	0.125	
CCL3L3	1.7	8.9	0.064	0.101	
CCL4	13.1	109.9	0.000	0.001	***
CCL4L1	2.2	28.4	0.001	0.003	**
CCL4L2	3.1	36	0.001	0.004	**
CCL5	26.7	73.3	0.003	0.008	**
CCL7	0.1	3.1	0.002	0.005	**
CCL8	1.9	22.4	0.000	0.000	***
CCL11	0.1	2.6	0.002	0.006	**
CCL13	13.3	24.4	0.113	0.159	
CCL14	15.2	16.5	0.447	0.514	
CCL15	0.1	2	0.002	0.007	**
CCL15–CCL14	0.1	0.3	---	---	
CCL16	1.2	1.1	0.595	0.656	
CCL17	1.1	2.2	0.111	0.159	
CCL18	54.5	520	0.000	0.001	***
CCL19	17	56.6	0.017	0.032	*
CCL20	0.3	1.5	0.009	0.021	*
CCL21	17.5	53.6	0.006	0.014	*
CCL22	5.9	12.9	0.079	0.122	
CCL23	0.8	2.9	0.016	0.030	*
CCL24	0	0.1	---	---	
CCL25	0	0	---	---	
CCL26	0.9	0.7	0.713	0.767	
CCL27	49.2	17	0.156	0.204	
CCL28	4.5	2.8	0.102	0.151	
CXCL1	3.3	49.4	0.001	0.003	**
CXCL2	1.9	4.9	0.002	0.006	**
CXCL3	0.1	0.7	0.040	0.068	
CXCL5	0	31.6	0.000	0.002	**
CXCL6	0	1.9	0.025	0.044	*
CXCL8	0.9	28.2	0.001	0.004	**
CXCL9	48.8	475.3	0.000	0.002	**
CXCL10	41.6	404.3	0.001	0.003	**
CXCL11	3.7	43.8	0.000	0.001	***
CXCL12	110.6	121.7	0.720	0.767	
CXCL13	1	15.1	0.009	0.019	*
CXCL14	446.4	158.1	0.113	0.159	
CXCL16	14.7	41.1	0.001	0.003	**
CXCL17	0.1	1.1	0.052	0.084	

*Differential expression between injected (*n* = 9) and uninjected (*n* = 10) were tested using a non-parametric two-sided Mann–Whitney test. Genes were considered significant at the 5% alpha threshold. Multiple hypothesis testing was accounted for using Benjamini–Hochberg false discovery correction*.

*Avg.Uninj, average uninjected; Avg.Inj, average injected; p.value, Mann–Whitney test p-value; p.adjusted, Benjamini–Hochberg adjusted p-value*.

Significance: *, <0.005; **, <0.01; ***, <0.001.

*---, gene was not evaluated because average FPKM levels across samples were less than 0.25*.

**Table 2:** **Expression levels of cytokines in bacille Calmette–Guérin injected and uninjected melanoma tissues**.

Cytokine name	avg.Uninj (10)	avg.Inj (9)	*p*.value	*p*.adjusted	Significance
GMCSF	0.2	1	0.019	0.035	*
IFNα	0	0	---	---	
IFNβ	0	0	---	---	
IFNγ	0.9	7.4	0.004	0.011	*
IL-1A	0.2	1.9	0.002	0.006	**
IL-1B	1	82.7	0.000	0.001	***
IL-1F10	1	0.3	0.281	0.353	
IL-2	0	0.2	---	---	
IL-3	0.1	0	---	---	
IL-4	0.1	0	---	---	
IL-5	0	0.1	---	---	
IL-6	1.1	20.2	0.002	0.006	**
IL-6ST	32.1	36.2	0.400	0.466	*
IL-7	1.6	2.6	0.050	0.082	
IL-8 (CCL8)	1.9	22.4	0.000	0.000	***
IL-9	0.1	0.1	---	---	
IL-10	1.8	3.5	0.006	0.014	*
IL-11	0.3	0.3	0.713	0.767	
IL-12B	0.1	0.4	0.016	0.030	*
IL-13	0.1	0.1	---	---	
IL-14	15.1	15.2	0.968	0.974	
IL-15	2	8.9	0.000	0.002	**
IL-16	7.6	6.1	0.133	0.177	
IL-17A	0	0	---	---	
IL-17B	0.1	0.3	---	---	
IL-17C	0	0	---	---	
IL-17D	6.9	0.8	0.002	0.006	**
IL-17F	0	0	---	---	
IL-18	39.4	42.1	0.400	0.466	
IL-18BP	12.2	45.5	0.001	0.003	**
IL-19	0	0.1	---	---	
IL-20	0	0.5	---	---	
IL-21	0	0.1	---	---	
IL-21-AS1	0	0.1	---	---	
IL-22	0	0.1	---	---	
IL-23A	0.1	0.8	0.007	0.016	*
IL-24	0.1	2	0.014	0.027	*
IL-25	0	0	---	---	
IL-26	0.1	0.7	0.001	0.004	**
IL-27	0	0.2	---	---	
IL-28	0	0	---	---	
IL-29	0.1	0	---	---	
IL-31	0	0	---	---	
IL-32	9.4	52.7	0.000	0.001	***
IL-33	7.8	13.1	0.243	0.310	
IL-34	4	1.5	0.315	0.387	
IL-36A	0	0.1	---	---	
IL-36B	1	1	0.902	0.919	
IL-36G	4.3	3.1	0.967	0.974	
IL-37	14.9	2.1	0.108	0.158	
TGFβ1	2.9	5.8	0.004	0.011	*
TGFβ2	7	1.5	0.065	0.103	
TGFβ3	6	11.8	0.028	0.048	*
TNFα	2.2	9.2	0.000	0.000	***
TNFβ	0.2	1.3	0.001	0.004	**

*Differential expression between injected (*n* = 9) and uninjected (*n* = 10) were tested using a non-parametric two-sided Mann–Whitney test. Genes were considered significant at the 5% alpha threshold. Multiple hypothesis testing was accounted for using Benjamini–Hochberg false discovery correction*.

*Avg.Uninj, average uninjected; Avg.Inj, average injected; p.value, Mann–Whitney test p-value; p.adjusted, Benjamini–Hochberg adjusted p-value*.

Significance: *, <0.005; **, <0.01; ***, <0.001.

*---, gene was not evaluated because average FPKM levels across samples were less than 0.25*.

### IL-BCG Upregulates Local Expression of Molecules That May Present Antigens to γδ T Cells, and BCG Stimulation Preferentially Activates and Expands Vγ9 T Cells

**Figure 3:**
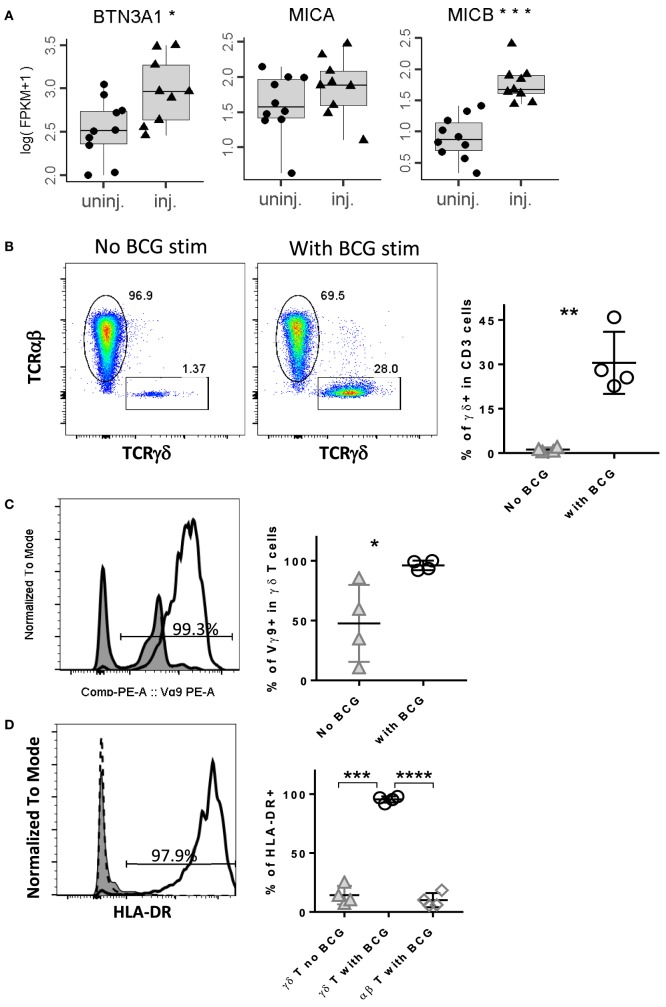
**IL-BCG injection induces the elevated expression of target antigens for γδ T cells, bacille Calmette–Guérin (BCG) stimulation preferentially activates and expands γ9δ2 T cells**. **(A)** IL-BCG induces the elevated expression of antigens for γδ T cells. **(B)** BCG stimulation expands γδ T cells preferentially. Left panel: histogram of FACS staining on γδ T cells without BCG stimulation for 6 days. Middle panel: histogram of FACS staining on γδ T cells with BCG stimulation for 6 days. Right panel: summary of percentage of γδ+ T cells without or with BCG stimulation for 6 days. **, paired *t*-test, *p* < 0.01. **(C)** BCG expanded γδ T cells are predominantly Vγ9+ cells. Left panel: a representative histogram of FACS staining on Vγ9. Right panel: summary of percentage of Vγ9+ cells in γδ T cells treated with or without BCG. *, paired *t*-test, *p* < 0.05. **(D)** BCG preferentially activates γδ T cells, not αβ T cells. Left panel: a representative histogram of FACS staining on HLA-DR. Right panel: summary of percentage of HLA-DR+ cells for γδ T cells with or without BCG stimulation and αβ T cells with BCG stimulation. ***, paired *t*-test, *p* < 0.001; ****, paired *t*-test, *p* < 0.0001.

Unlike αβ T cells that recognize peptide fragments restricted on MHC molecules, γδ T cells recognize unconventional antigens such as normal cellular metabolites of IPP presented on BTN3A1 (CD277) molecules (19, 22) or the stress-induced MHC class-I chain-related molecules, MICA and MICB (45). To investigate whether IL-BCG injection induces these γδ T cell targets, we compared the RNA expression profiles between IL-BCG non-injected and injected lesions. IL-BCG injection induced the elevated expression of both BTN3A1 and MICB molecules (Figure 3A).

To examine whether BCG could activate and expand γδ T cells, we stimulated the PBMCs with live BCG. After 6 days of *in vitro* stimulation, γδ T cells were significantly expanded in the cultures treated with BCG, but not in the wells without BCG (Figure 3A). The newly expanded γδ T cells expressed a high level of HLA-DR, an activation marker for T cells after they respond to the cognate antigen stimulation (Figure 3D, solid line). Little or no HLA-DR expression was seen on γδ T cells in the well without BCG (Figure 3D, shaded area). Also, little or no HLA-DR expression was seen on αβ T cells from the well treated with BCG (Figure 3D, dashed line). The expanded cells were predominantly Vγ9-expressing cells (Figures 3B,C). Together, these data suggest that BCG preferentially activates γδ T cells, specifically, Vγ9-expressing cells.

### γδ T Cells Infiltrate Uninjected Regressed Tumor Lesions

**Figure 4:**
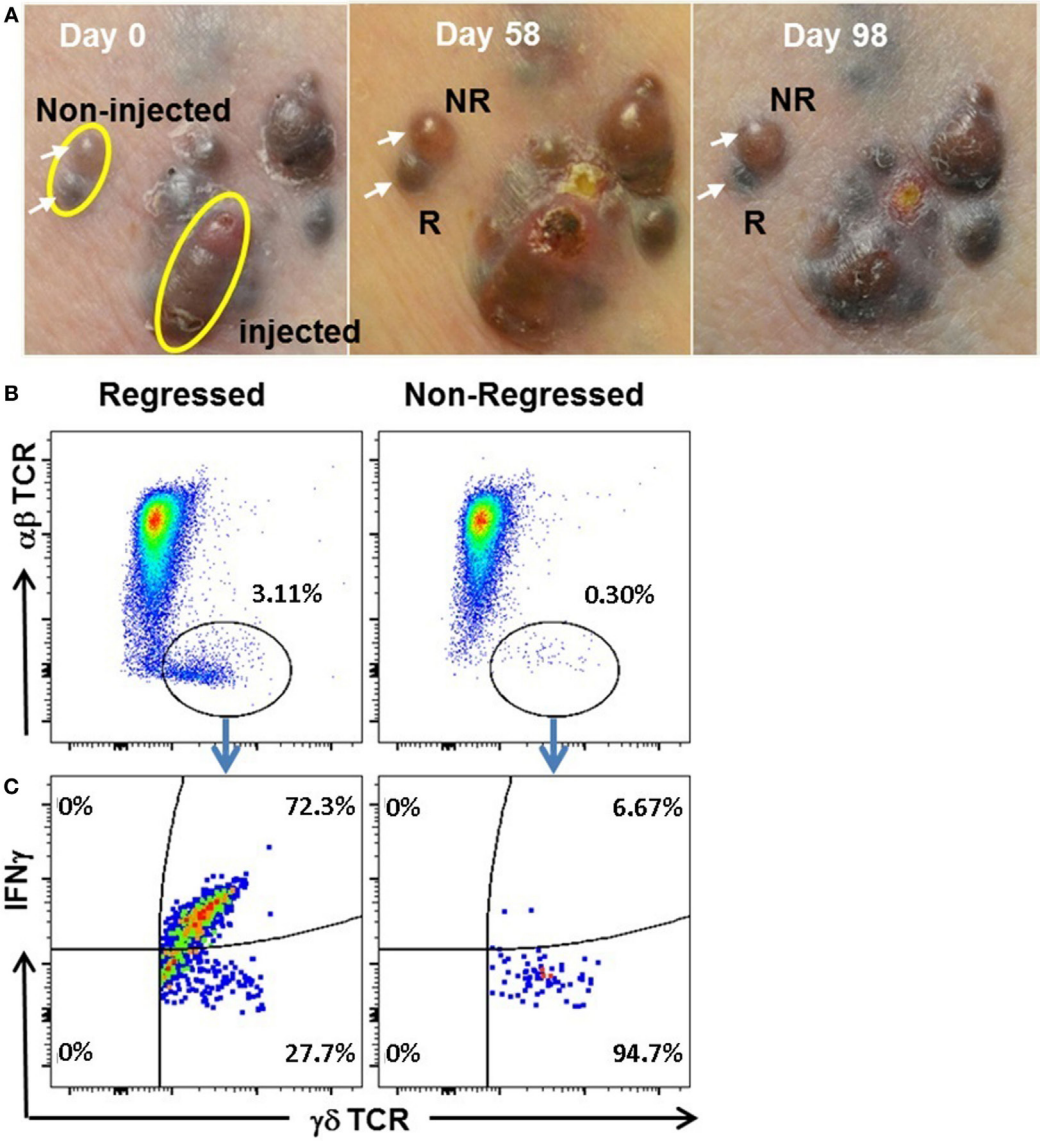
**IL-BCG induces tumor regressions in uninjected melanoma lesions**. **(A)** IL-BCG induces regression of uninjected lesions. Upper left: in-transit melanoma metastases at the time of first IL-BCG treatment. Upper middle: 8 weeks post-IL-BCG, tumors exhibit mild to moderate inflammation. Upper right: 13 weeks post-IL-BCG. Adjacent uninjected tumors (white arrows) exhibiting regression (R) or non-regression (NR). **(B)** Presence of γδ T cells in regressed but not non-regressed uninjected melanoma lesions. **(C)** IFNγ secretion of tumor infiltrated by γδ T cells. Tumors infiltrated by γδ T cells secrete IFNγ isolated from regressed but not non-regressed uninjected melanoma lesions.

IL-BCG treatment not only induces the regression of injected tumors but also occasionally induces the regression of uninjected tumors (Figure 4A) (5, 6). To investigate whether γδ T cells also participate in the regression of non-injected in-transit lesions during IL-BCG treatment, we compared TILs from regressed and non-regressed tumors. Both lesions studied had not been injected with BCG (Figure 4A). After 21 days of culture in the presence of IL-2 and IL-15, the composition of TILs from regressed and non-regressed tissues was analyzed *via* flow cytometry. Figure 4B shows a clear population of γδ T cells in the TILs from the regressed melanoma tissue. Interestingly, 72.3% of these γδ T cells from regressed melanoma specimens express IFNγ in this mixed culture of tumor and TILs without additional stimulation (Figure 4C). No such population of γδ T cells was found in TILs from adjacent non-regressed melanoma tissue (Figure 4C). Similar analysis was performed on the αβ T cell population. αβ T cells from both regressed and non-regressed tissues secreted minimal levels (0.07 and 0.12%, respectively) of IFNγ (Figure S2 in Supplementary Material). These data suggest that IFNγ from γδ T cells may contribute to tumor regression by IL-BCG.

## Discussion

Bacille Calmette–Guérin has been widely used as an immunotherapeutic adjuvant for over 30 years (5, 6). Currently, it is a recommended agent for the treatment of bladder cancer and melanoma in the NCCN guidelines. Despite this, the mechanisms of BCG responses are poorly understood. Previous studies have revealed that multiple cellular components and soluble factors contribute to the BCG-induced antitumor activity of bladder cancer (7), including T cells, natural killer cells, macrophages, dendritic cells, and even granulocytes, as well as the soluble factors released from these cells. In clinical trials, immune competence is also a prerequisite of BCG-induced antitumor activity in melanoma (5, 6). Animal studies highlight that CD4 and CD8 T cells, in addition to NK cells, played indispensable roles (7). Lack of these cells abolished BCG-induced antitumor activity of BCG vaccination in bladder cancer in animal models (7, 46, 47). Therefore, it is likely that adjuvant therapy with BCG induces multilayer antitumor activities. Discovering what is behind these antitumor activities is essential to optimizing the therapeutic effects of adjuvant BCG therapy. Few studies have focused on the role played by γδ T cells in BCG-induced antitumor activity (35). γδ T cells comprise a small proportion of T cells that have limited T cell receptor diversity and unconventional, MHC-independent, antigen-recognition mechanisms. The role of γδ T cells in tumor immunology has not been fully appreciated when compared to αβ T cells. Here, we found that IL-BCG treatment induces a response that has the capacity to actively attract γδ T cells, selectively expand Vγ9 T cells, and upregulate antigens recognized by Vγ9Vδ2 T cells in injected lesions. These data suggest that γδ T cells are strongly associated with the antitumor activity induced by IL-BCG. Furthermore, γδ T cells are also present in regressed lesions that are not injected with BCG. This association suggests a primary role of γδ T cells in the induction of melanoma regressions (48).

It has been reported that γδ T cells play a critical role in the control of mycobacterial infections in both humans and animals (49, 50). Primary *M. bovis* BCG infection induces major clonal expansion of phosphoantigen-specific Vγ2Vδ2 (also termed as Vγ9Vδ2) T cells (51, 52). In this study, we confirmed that BCG preferentially activates and expands Vγ9 γδ T cells in PBMC *in vitro*, with more than a 20-fold expansion in 6-day stimulation from frozen PBMCs (Figure 3B). Meanwhile, very few CD3+ αβ T cells were activated and expanded from these PBMCs (Figure 3D). This rapid, preferential activation of Vγ9 γδ T cells is not only consistent with the role γδ T cells play in the early control of mycobacterial infections (9, 10) but also provides a mechanism by which Vγ9Vδ2 T cells could impact antitumor activity (38, 48).

Target antigens for Vγ9Vδ2 T cells are phosphoantigens, the low molecular weight metabolites of the mevalonate pathway (19, 22). Infection and physiologic stress can cause dysregulation of the mevalonate pathway and induce elevated phosphoantigen metabolism. Meanwhile, infection and stress also induce expression of the BTN3A family of molecules, putative molecules that present phosphoantigens to Vγ9Vδ2 T cells (22). It is not surprising, therefore, that IL-BCG treatment can induce melanoma regression at injected sites, and that these regressions are strongly associated with the presence of Vγ9Vδ2 T cells (48). Besides infection, tumors are stressed due to rapid growth, limited oxygen and nutrient supplies. These physiologic stressors also induce dysregulation of the mevalonate pathway and expression of BTN3A proteins (22). Since BCG preferentially activates and expands Vγ9Vδ2 T cells in a locoregional manner, it is perceivable that some Vγ9Vδ2 T cells could migrate to the stressed tumor itself and execute antitumor activity in non-injected lesions. In addition to mediating the expression of BTN3A for γδ T cell response, BCG therapy also enhances the expression of MIC B in injected tumor lesions (Figure 3A), another target Ag for γδ T cells and NK T cells (7). The main point of our study was to investigate the role of γδ T cells on the activity induced by IL-BCG treatment but was not intended to exclude the potential role of other cells. We cannot rule out that NK cells may also contribute to the observed regression, and future studies are warranted to determine the mechanism of regression to answer whether γδ T cells actively lyse the tumors or influence the tumor cells to limit growth in other ways. Since NK cells are known to have these capabilities, their potential role in BCG cannot be ruled out.

Bacille Calmette–Guérin infection or vaccination induces strong delayed type hypersensitivity responses (7), a typical Th1 type response. This response recruits diversified inflammatory cells and stimulates the release of a variety of cytokines and chemokines. IL-BCG treatment induces a long-lasting high-level release of chemokines such as CXCL9, 10, and 11, a set of chemokines that recruit CXCR3 expression cells. Interestingly, 90% of γδ T cells in the peripheral blood express high levels of CXCR3 receptors. This observation further supports the conclusion that IL-BCG can recruit γδ T cells to the injected lesions and expand the Vγ9Vδ2 T cell subset. This observation further supports the conclusion that IL-BCG can recruit γδ T cells to the injected lesions and expand the Vγ9Vδ2 T cell subset.

Cytokines are the mediators of immune cells that execute the activity of immune responses. Among the long list of cytokines expressed at BCG-injected tumor lesions (Table 2), the expression of several cytokines, IFNγ (42, 43), IL-15 (44), and TNFα, accompany the increase in γδ T cells. These cytokines can have direct and indirect antitumor activities during immune responses (7). Therefore, expression of these cytokines (despite the fact that they are not unique for γδ T cells and can be expressed by NK cells and other αβ T helper cells) also corroborates the hypothesized positive role of γδ T cells in BCG-induced antitumor activity. Alternately, BCG is live vaccine that can induce strong cellular immune responses. As negative feedback of these immune responses, it is expected that BCG injection will induce the expression of regulatory factors, such as immunosuppressive cytokines that can downregulate antitumor activity. IL-17 is a product of regulatory γδ T cells during the early phase of immune response (53, 54). IL-17-producing regulatory γδ T cells have been shown to mediate pro-tumor activities in mouse models (53, 54). Interestingly, the expression of IL-17 (only IL-17D isoform) is clearly downregulated in BCG-injected melanoma lesions (Table 2). It is possible that the time of sample collection is out of IL-17 expression phase, or BCG recruited γ9δ2 subset is effector γδ T cells (53–55). Nevertheless, this observation also supports the notion of a positive role of γδ T cells in BCG-induced antitumor activity. However, BCG injection does induce the expression of cytokines that play pro-tumor activities, including IL-1β, 6, 8, 10, 18BP (56), 26, 32 (57), and TGFβ (58, 59) (Table 2). Whether all or part of these cytokines are produced by γδ T cells in BCG-injected lesions is unknown. This will be an important topic for the future study to improve the efficacy of BCG therapy, since BCG therapy does not always eradicate melanoma completely.

IL-BCG therapy can induce regression of injected and uninjected melanoma lesions, but durable clinical responses are not observed in all treated patients. The limited effect of this treatment may be due to the transient nature of the inflammatory response induced. We found the highest expression of chemokines and cytokines in BCG-treated melanoma skin metastases 4 weeks after IL-BCG injection are CCL2 (40.8 vs. 157.1, of the average FPKM in BCG uninjected vs. BCG injected melanoma tissues), CCL3 (12.4 vs. 75.7), CCL4 (13.1 vs. 109.9), CCL5 (26.7 vs. 73.3), CCL18 (54.5 vs. 520), CXCL9 (48.8 vs. 475.3), and CXCL10 (41.6 vs. 404.3) (Table 1). These chemokines are usually released from the monocyte/macrophage lineage and mast cells during the acute phase of the inflammatory immune response (60, 61). High-level expression of these chemokines not only recruits and activates type 1 macrophages and neutrophils (60) but also recruits immune regulatory cells such as T regulatory cells through CCL2, CCL18 chemotaxis (60, 62, 63), myeloid-derived suppressor cells (60, 62, 63), and type 2 neutrophils (60, 64) through CCL2, CCL3, CCL4, and CCL5 chemotaxis. These immunosuppressive cells may counter any antitumor immune responses. On the other hand, high-level expression of CXCL9 and CXCL10 recruit Th1 CD4 cells, cytotoxic CD8 T cells, NK cells, and γδ T cells, through the CXCL9 and CXCL10/CXCR3 chemotactic axis (60). Activation of these cells can stimulate IFNγ, IL-6, IL-8, IL-10, IL-15, IL-18BP, IL-32, TGFβ1, TGFβ3, and TNFα release as the average FKPMs of most of cytokines are below 10 (Table 2) in contrast to 100–500 for the chemokines (Table 1). Whatever the source, a substantial number of cytokines induced by IL-BCG favor the tumor, such as IL-1β (65), IL-6 (66), IL-8 (67), IL-10, IL-18BP (65), IL-32 (57, 68), and TGFβ (55, 56). Even TNFα and IFNγ may have dual effects. For example, at high concentrations, TNFα can kill sarcoma cells by binding to the TNFR1 and inducing apoptosis (58). In contrast, at sustained low levels, TNFα promotes tumorigenesis by inducing the generation of reactive oxygen and nitrogen species to induce DNA damage (58). IFNγ can activate cytotoxic T cells and macrophages to kill tumor cells; meanwhile, persistent expression of IFNγ can induce expression of immune checkpoint molecules such as PD-L1 on tumor cells and antigen-presenting cells to diminish T cell antitumor activities (69). How the combination of cytokines, chemokines, and the cells they recruit to the local tumor microenvironment lead to tumor progression or regression has yet to be understood and likely depends on numerous factors that vary from individual to individual. BCG, as an attenuated live vaccine, is an excellent biological agent to investigate the effect of local inflammation on antitumor activity. Our studies indicate γδ T cells may play an important role in this process.

In summary, our study suggests an association of Vγ9Vδ2 T cells with IL-BCG-induced melanoma regressions, consistent with the latest clinical discovery that the frequency of Vδ2+ γδ T cells is positively correlated with overall survival in melanoma patients treated with ipilimumab (48). Analysis of gene expression profiles induces type 1 immunity and may promote a unique Vγ9Vδ2 T cell infiltration profile. Further study of these immunological profiles will provide valuable insight in the search for novel checkpoint targets and for the development of novel combinations of BCG therapy and checkpoint inhibition in melanoma and bladder cancer.

## Ethics Statement

Alpha IRB approved this study. All participants meeting inclusion criteria were given informed consent. The treating physician invited patients who met the inclusion/eligibility criteria. Patients were given the consent forms at the physician’s office. They were welcomed to review the forms at home and had an opportunity to discuss their participation in the research with whomever they wished. The study coordinator/nurse and treating physician made certain the subject understood the research plan and objective as well as the risks and benefits of the study. The physician questioned the prospective subject about the research and was available for questions. No information was withheld from the subject. No vulnerable populations were involved in this study.

## Author Contributions

JY: experimental design and performance as well as manuscript writing. MJ: manuscript preparation, collection of patient and treatment information, manuscript writing, and provision of critical considerations for experiment design. AC: performance of bioinformatics and statistical analysis and manuscript editing. AL and RR: experiment performance. PS: experimental design and manuscript revision. LF and MF: patient recruitment and treatment, manuscript revision, and provision of critical considerations for experiment design. DL: study oversight, patient recruitment, experimental design, manuscript writing, and provision of critical considerations for experiment design.

## Conflict of Interest Statement

The authors declare that the research was conducted in the absence of any commercial or financial relationships that could be construed as a potential conflict of interest.

## Supplementary Material

Figure S1 **IL-BCG induces expression of CCL22 and CCL20**. **(A)** IL-BCG induces upregulation of CCL22 and CCL20; **(B)** expression of CCR4. γδ T cells from PBMC do not express CCR4; **(C)** expression of CCR6. γδ T cells from PBMC do not express CCR6.

Figure S2 **IFNγ secretion of tumor infiltrated by αβ T cells**. Tumor infiltrated by αβ T cells do not secrete IFNγ isolated from either regressed or non-regressed, uninjected melanoma lesions.
