# Supplementary material for: Insights into Local Tumor Microenvironment Immune Factors Associated with Regression of Cutaneous Melanoma Metastases by Mycobacterium bovis Bacille Calmette–Guérin
Source: Front Oncol. 2017 Apr 5;7:61. doi: 10.3389/fonc.2017.00061 (PMC5380679; doi:10.3389/fonc.2017.00061)
Supplement: Supplementary file 1 [file table_1.pdf]

Supplemental Table 1. Patient and Biopsy Information.

Patient Number	Sex	Age at Diagnosis	Breslow Thickness of Primary	Response to ILBCG	Number of uninjected biopsies	Number of BCG injected biopsies	Weeks post ILBCG Injection
1	M	77	2.6 mm	PR	1†	1†	9
3	F	84	1.5mm	NR		2	6, 8
4	F	62	1.95mm	R	2	1	4
5	M	67	1.75mm	R	1	5	4, 9,14,14,14
6	M	62	1.75mm	R		1	4
7	F	85	>4.5mm	NR	4		
9	F	85	2.2mm	R	3		
10	F	72	3.2 mm	R	1‡	1‡	14

R: responder

NR: Non-responder

PR: partial responder

†: tissue for immunohistochemistry

‡: tissue for TIL isolation
