# Supplementary figures and images for: Insights into Local Tumor Microenvironment Immune Factors Associated with Regression of Cutaneous Melanoma Metastases by Mycobacterium bovis Bacille Calmette–Guérin
Source: Front Oncol. 2017 Apr 5;7:61. doi: 10.3389/fonc.2017.00061 (PMC5380679; doi:10.3389/fonc.2017.00061)

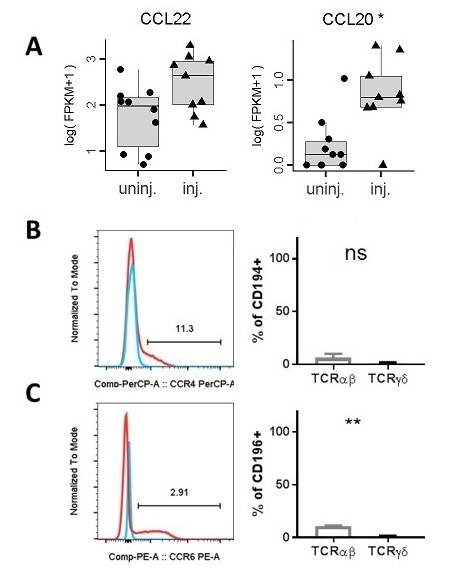

Supplement: Figure S1 — IL-BCG induces expression of CCL22 and CCL20. (A) IL-BCG induces upregulation of CCL22 and CCL20; (B) expression of CCR4. γδ T cells from PBMC do not express CCR4; (C) expression of CCR6. γδ T cells from PBMC do not express CCR6. [file image_1.jpeg]

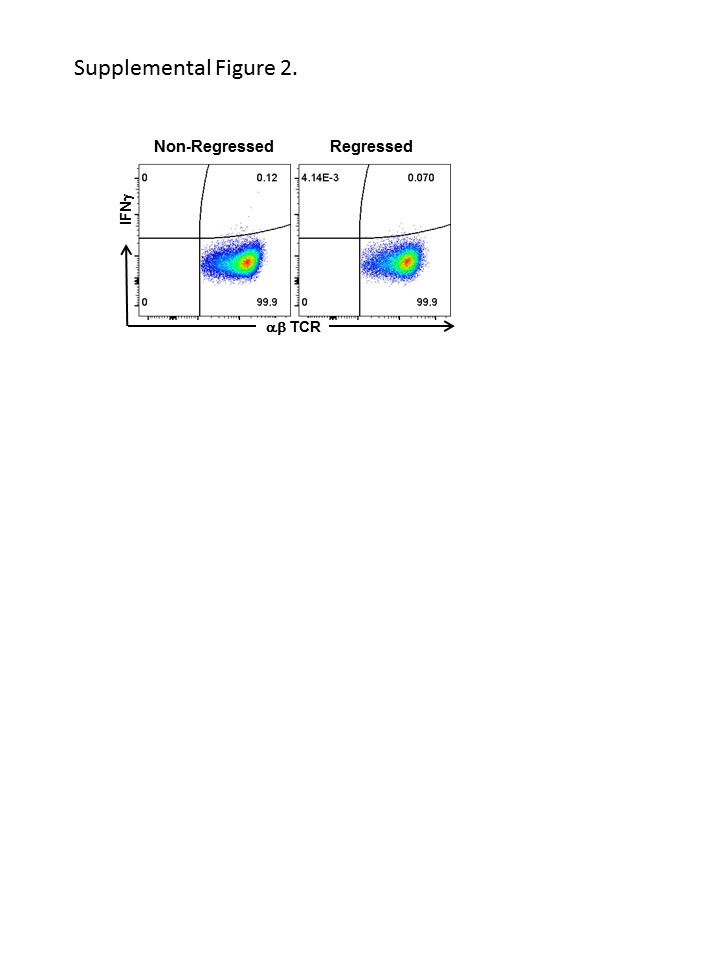

Supplement: Figure S2 — IFNγ secretion of tumor infiltrated by αβ T cells. Tumor infiltrated by αβ T cells do not secrete IFNγ isolated from either regressed or non-regressed, uninjected melanoma lesions. [file image_2.jpeg]
